# Peculiarities of the Variation of Biologically Active Compounds in Fruit of *Vaccinium oxycoccos* L. Growing in the Čepkeliai State Strict Nature Reserve

**DOI:** 10.3390/molecules28155888

**Published:** 2023-08-05

**Authors:** Rima Šedbarė, Onutė Grigaitė, Valdimaras Janulis

**Affiliations:** 1 Department of Pharmacognosy, Faculty of Pharmacy, Lithuanian University of Health Sciences, 50162 Kaunas, Lithuania; valdimaras.janulis@lsmu.lt; 2 Dzūkija National Park and Čepkeliai State Nature Reserve Directorate, 65334 Merkinė, Lithuania

**Keywords:** bog cranberry, UPLC, flavonoids, triterpenoids, procyanidins, anthocyanins

## Abstract

This study was carried out to analyze the accumulation patterns of anthocyanins, proanthocyanidins, flavonols, chlorogenic acid, and triterpene compounds in fruit samples of *Vaccinium oxycoccos* L. berries growing in the Čepkeliai State Strict Nature Reserve in Lithuania. Studies were carried out on the phytochemical composition of cranberry fruit samples during the period of 2020–2022. Anthocyanins, flavonols, chlorogenic acid and triterpene compounds were identified and quantified using UPLC-DAD methods, and proanthocyanins were determined using spectrophotometric methods. The content of identified compounds varied, as reflected in the total amounts of anthocyanins (710.3 ± 40 µg/g to 6993.8 ± 119 µg/g), proanthocyanidins (378.4 ± 10 µg EE/g to 3557. 3 ± 75 µg EE/g), flavonols (479.6 ± 9 µg/g to 7291.2 ± 226 µg/g), chlorogenic acid (68.0 ± 1 µg/g to 3858.2 ± 119 µg/g), and triterpenoids (3780.8 ± 98 µg/g to 7226.9 ± 224 µg/g). Cranberry fruit samples harvested from open oligotrophic wetland habitats contained higher levels of anthocyanins, anthocyanidins, flavonol glycosides, and proanthocyanidins. The highest levels of triterpene compounds were found in the cranberry fruits harvested in the spring of the following year after the snowmelt. The use of principal component analysis showed that cranberry plant material harvested in October and November had higher levels of bioactive compounds.

## 1. Introduction

The number of wetlands is declining worldwide, and their ecosystems are fragile: more than 50% of specific types of wetlands in parts of North America, Europe, Australia, and New Zealand were destroyed during the twentieth century [1]. Destroyed wetlands and drained peatlands release gases that increase the greenhouse effect in the Earth’s atmosphere. Thus, preserving and restoring wetlands is important in efforts to reverse climate change and address atmospheric stability issues [2]. In order to preserve sensitive wetland ecosystems in Lithuania, the Čepkeliai, Kamanai, and Viešvilė State Strict Nature Reserves and the Žuvintas State Biosphere Reserve have been established [3]. The Čepkeliai wetland is one of the largest and most unique natural raised bogs (5858 hectares of raised bogs) in the Baltic region, containing rare and endangered plant species and populations of protected animals [4,5]. The research carried out in the Čepkeliai State Strict Nature Reserve is important for assessing the changes in wetlands in a warming climate, for assessing their status, and for developing strategies to conserve wetland ecosystems and restore damaged or destroyed wetlands in Lithuania [3,6].

In the reserves and strict reserves located in the territory of Lithuania, human visitation and economic activities are strictly limited. Phytochemical studies into cranberry fruit are important as they determine the optimum time for harvesting cranberries and provide research-based recommendations for their rational harvesting. The results of research and the implementation of recommendations based on research data would allow the Lithuanian Reserve Authorities to set time limits for the issuing of permits for the harvesting of cranberries by the population in protected areas, justify the procedure for issuing permits and optimize the use of natural resources for the population. The ripening of cranberries results in the accumulation of the highest levels of biologically active compounds, and thus studies on the qualitative and quantitative composition of the cranberry fruit are important for assessing the quality of raw plant material.

The results of studies on the phytochemical composition of *Vaccinium oxycoccos* fruits are rather fragmentary [7]. The composition of bog cranberry fruits was investigated using the spectrophotometric method [8,9] and liquid chromatography methodology with diode array detectors and mass spectrometry detectors [10,11,12]. Fresh cranberry fruit samples were found to contain anthocyanins (12.4–207.3 mg/100 g), proanthocyanidins (1.5–5.3 mg/100 g), flavonols (36.3 mg/100 g), phenolic acids (96.3 mg/100 g), vitamin C (31 mg/100 g), and triterpene compounds (12.9 mg/100 g) [7,10]. The variety of bioactive compounds and their biological effects makes cranberries a promising medicinal plant material.

The cultivation of cranberries and the rational use of natural cenopopulations for their harvesting and storage are promising for the development of agro-friendly farming [13,14]. Chemical composition studies are important and provide knowledge on the use of *Vaccinium oxycoccos* fruits as alternatives to *Vaccinium macrocarpon* fruits due to their similar flavor characteristics and a similar composition of bioactive compounds [7,9]. A detailed investigation into the bioactive compound content of cranberry fruits can help to protect cranberry habitats, provide a rationale for the use of cranberry resources, and guarantee the preparation of quality cranberry raw material.

The aim of the investigation was to perform a phytochemical composition analysis on the fruits of *Vaccinium oxycoccos* growing in the habitats of the Čepkeliai State Strict Nature Reserve. In order to determine the variation in the qualitative and quantitative composition of triterpene, chlorogenic acid, flavonols, proanthocyanidins, and anthocyanins compounds during ripening, studies were carried out in the period from 2020 to 2022 on *Vaccinium oxycoccos* cenopopulations. The obtained data are relevant for determining the optimal time of cranberry harvesting, providing recommendations for the rational harvesting of cranberry fruits, and clarifying the influence of different wetland habitats on the phytochemical composition of cranberry fruits.

## 2. Results and Discussion

### 2.1. Analysis of the Qualitative and Quantitative Composition of Anthocyanins

Fresh and processed cranberry fruits are used in food supplements, juice production, and in the food industry. Thus, anthocyanin studies on fruit samples of bog cranberries growing in Lithuania are relevant for assessing the quality and health benefits of cranberry fruit raw material.

**Figure 1:**
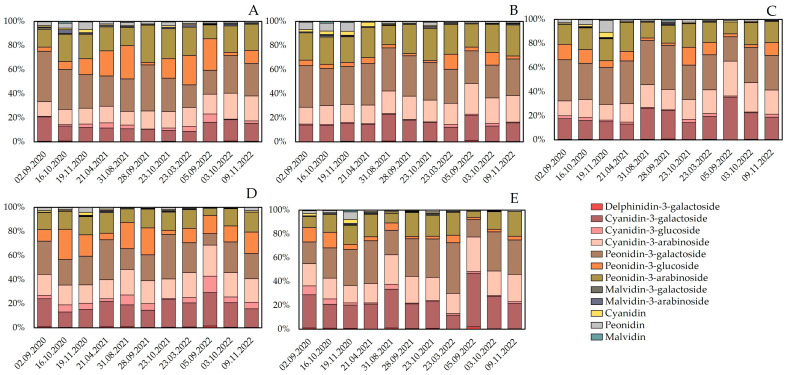
Percentage distribution of identified anthocyanins and anthocyanidins in cranberry fruit samples harvested from different wetland habitats. Descriptions of wetland habitats marked with different letters (**A**–**E**) are given in Table 1.

**Figure 2:**
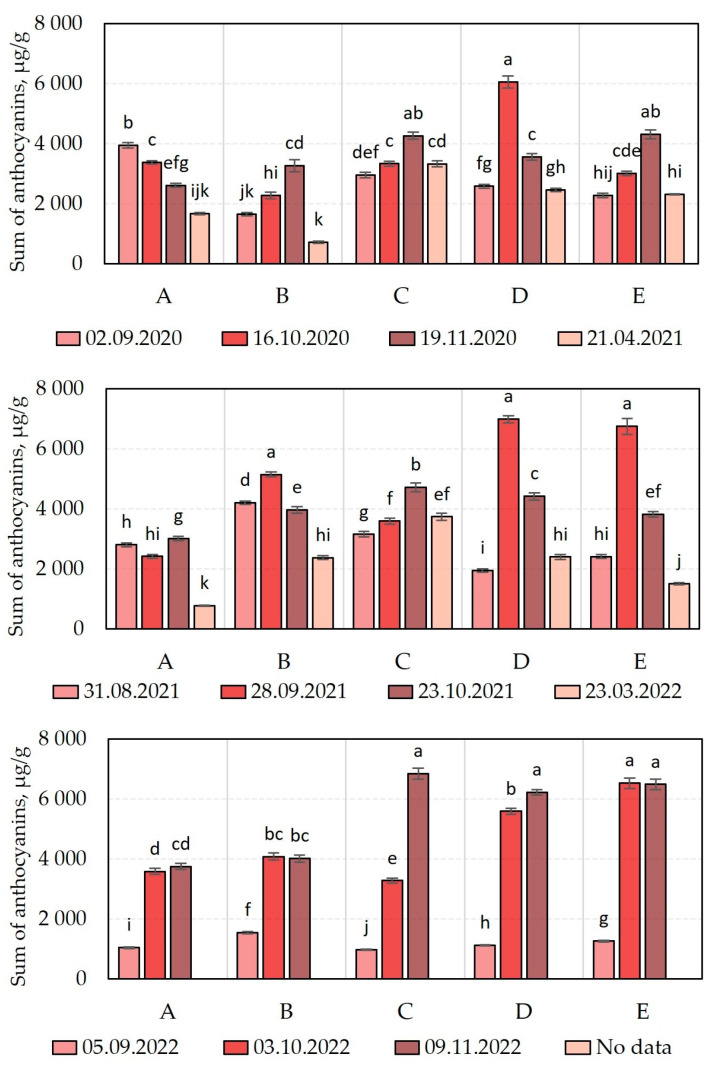
Amount of anthocyanins in cranberry fruit samples. The wetland habitats (**A**–**E**) of the cranberry fruit sampling sites are described in Table 1. Alphabetic letters indicate the significant difference (*p* < 0.05) between the sum of anthocyanins in the tested cranberry fruits.

The qualitative and quantitative composition of anthocyanins and anthocyanidins in bog cranberry fruit samples harvested during different growing seasons are presented in Figure 1 and Figure 2. While the qualitative profile of anthocyanins in the phytochemical composition of bog cranberry (*Vaccinium oxycoccos*) fruit growing under Lithuanian climatic conditions was identical, the quantitative profile of anthocyanins in the cranberry fruit samples harvested from different types of habitat varied. Four anthocyanins were predominant in the cranberry fruit samples harvested from mesotrophic wetland (site B) and oligotrophic wetland (sites C and E) habitats: cyanidin-3-*O*-galactoside (20.1% ± 7.2%), cyanidin-3-*O*-arabinoside (19.0% ± 4.19%), peonidin-3-*O*-galactoside (30.4% ± 6.12%), and peonidin-3-*O*-arabinoside (18.4% ± 5.3%) (Figure 1). Meanwhile, six anthocyanins were prevalent in the fruit samples harvested from the mesotrophic wetland (site A) and the oligotrophic wetland (site D) habitats: cyanidin-3-*O*-galactoside (16.2% ± 5.1%), cyanidin-3-*O*-arabinoside (17.0% ± 3.7%), peonidin-3-*O*-galactoside (26.4% ± 7.5%), peonidin-3-*O*-arabinoside (17.4% ± 5.4%), cyanidin-3-*O*-glucoside (3.9% ± 3.1%), and peonidin-3-*O*-glucoside (14.4% ± 8.2%) (Figure 1). The analysis of the qualitative and quantitative composition of anthocyanins in bog cranberries growing in the territories of the Žuvintas and Kamanai State Strict Nature Reserves in Lithuania showed that the percentage of anthocyanins in cranberry fruit samples varied depending on the location of the plant. Four anthocyanins were predominant in cranberry fruit samples harvested from different wetland habitats (cyanidin-3-*O*-galactoside, cyanidin-3-*O*-arabinoside, peonidin-3-*O*-galactoside, and peonidin-3-*O*-arabinoside), while six anthocyanins predominated in others (cyanidin-3-*O*-galactoside, cyanidin-3-*O*-arabinoside, peonidin-3-*O*-galactoside, peonidin-3-*O*-arabinoside, cyanidin-3-*O*-glucoside, and peonidin-3-*O*-glucoside) [15].

Research has shown that cranberry genotype influences the quantitative composition of anthocyanins in cranberry fruit samples [16]. Areškevičiūtė et al. investigated the genetic properties of morphologically distinct bog cranberries (*Vaccinium oxycoccos*) using random amplified polymorphic DNA (RAPD) analysis and found a high level of genetic variability in the bog cranberry population [17]. Vosra et al. found that cranberry ploidy 2× and 4× affected the composition of anthocyanins glucosides, galactosides, and arabinosides in cranberry fruit [16]. Česonienė et al. reported that the anthocyanin composition profile of tetraploid-type *V. oxycoccos* fruit samples was similar to that of *V. macrocarpon* fruit samples [18]. Higher levels of cyanidin and peonidin glucosides were detected in the fruit samples of diploid-type cranberries [18]. In our study, the percentage of anthocyanins in the cranberry fruit samples harvested from different habitats varied between their ripening and the 3-year study period. The variation in anthocyanin percentage could be explained by the fact that different cranberry genotypes may grow in different habitats and that their variation from one habitat to another determines the qualitative and quantitative anthocyanin composition of the raw plant material. 

We carried out a comparative analysis of the total anthocyanin content in *V. oxycoccos* fruit samples harvested during the 2020–2022 growing season (Figure 2). We found that the total anthocyanin content varied among the fruit samples harvested: 710.3 ± 40 µg/g to 6054.5 ± 200 µg/g in the 2020 growing season, 779.0 ± 12 µg/g to 6993.8 ± 119 µg/g in 2021, and 974.8 ± 23 µg/g to 6840.9 ± 185 µg/g in 2022 (Figure 2). The anthocyanin content of cranberry fruit samples harvested in 2021 in the Kamanai and Žuvintas State Strict Nature Reserves varied over a wider range, from 698 ± 24 µg/g to 8352 ± 200 µg/g [15].

An increasing trend in the quantitative composition of anthocyanins was observed in cranberry fruit samples harvested during berry ripening from September to October in different years (2020–2022) in the habitats of the Čepkeliai State Strict Nature Reserve (Figure 2). We found that the anthocyanin content of the cranberry samples harvested in the second half of October and in November varied unevenly depending on the year and the type of the habitat (Figure 2). In cranberry fruit samples harvested after winter, the anthocyanin content showed a statistically discernible decrease (Figure 2).

The study of the anthocyanins levels in the cranberry fruits harvested in 2020 found the maximum observed total anthocyanin content (6054.5 ± 200 µg/g) in the cranberry fruits harvested on October 16 from the oligotrophic wetland habitat (site D). The anthocyanin levels of the fruits harvested from this site were not statistically discernible from the anthocyanin content levels of the cranberry fruit samples harvested from the oligotrophic wetland habitats (sites C and E) on November 19. The lowest levels of total anthocyanin (710.3 ± 40 µg/g) were found in the cranberry fruits harvested from the mesotrophic wetland habitat (site B) in the spring of the following year, after the snowmelt (on 21 April 2021).

In cranberry fruit samples harvested during the growing season of 2021, the highest total anthocyanin content (6993.8 ± 119 µg/g) was detected in samples harvested from an oligotrophic wetland habitat (site D) on September 28. The content of anthocyanins in the fruits harvested from this habitat was not discernibly statistically different from the anthocyanin content in the cranberry fruits harvested from the oligotrophic wetland habitat (site E) or the mesotrophic habitat (site B) on September 28. The lowest levels of total anthocyanins (779.0 ± 12 µg/g) were found in the cranberry fruits harvested from the mesotrophic habitat (site A) in the spring of the following year, after the snowmelt (on 23 March 2022).

The analysis of the quantitative composition of anthocyanins in cranberry fruits harvested in 2022 showed that cranberry fruits harvested on November 9 from an oligotrophic wetland habitat (site C) had the highest total anthocyanin content (6840.9 ± 185 µg/g). The levels of anthocyanin in these fruits were not discernibly statistically different from the anthocyanin content of the cranberry fruits harvested from the oligotrophic wetland habitat (sites E and D) on November 9. The lowest levels of total anthocyanin (974.8 ± 23 µg/g) during the 2022 growing season were found in cranberry fruits harvested from the oligotrophic wetland habitat (site C) in September.

According to the literature, anthocyanins are among the most important groups of biologically active compounds identified in cranberry fruit samples, possessing a range of disease-preventing and health-promoting effects [19]. The anthocyanin content of cranberry fruit samples increased, on average, by a factor of 2–4 in different years during the ripening period (September to October), making the timing of cranberry picking one of the most important factors influencing the qualitative and quantitative composition of anthocyanins in cranberry plant material. Studies on the qualitative and quantitative composition of anthocyanins are important for assessing the quality of cranberry plant material and for the development and production of healthy and organic cranberry products with a known composition.

### 2.2. Analysis of the Quantitative Composition of Proanthocyanidins

Proanthocyanidins detected in the bioactive compound matrix of cranberry fruit samples determine the effects of the fruit on the health of the urinary tract [20]. Proanthocyanidin dimers, trimers, oligomers, and polymers have been identified in cranberry fruit. A spectrophotometric method (the DMAC assay) is often used for the determination of the proanthocyanidin content in cranberry plant material and preparations derived from it. This spectrophotometric method is considered to be the fastest and most accurate method for the determination of the total amount of proanthocyanins in cranberry fruit samples, taking into account all the degrees of polymerization of proanthocyanidins [21,22].

**Figure 3:**
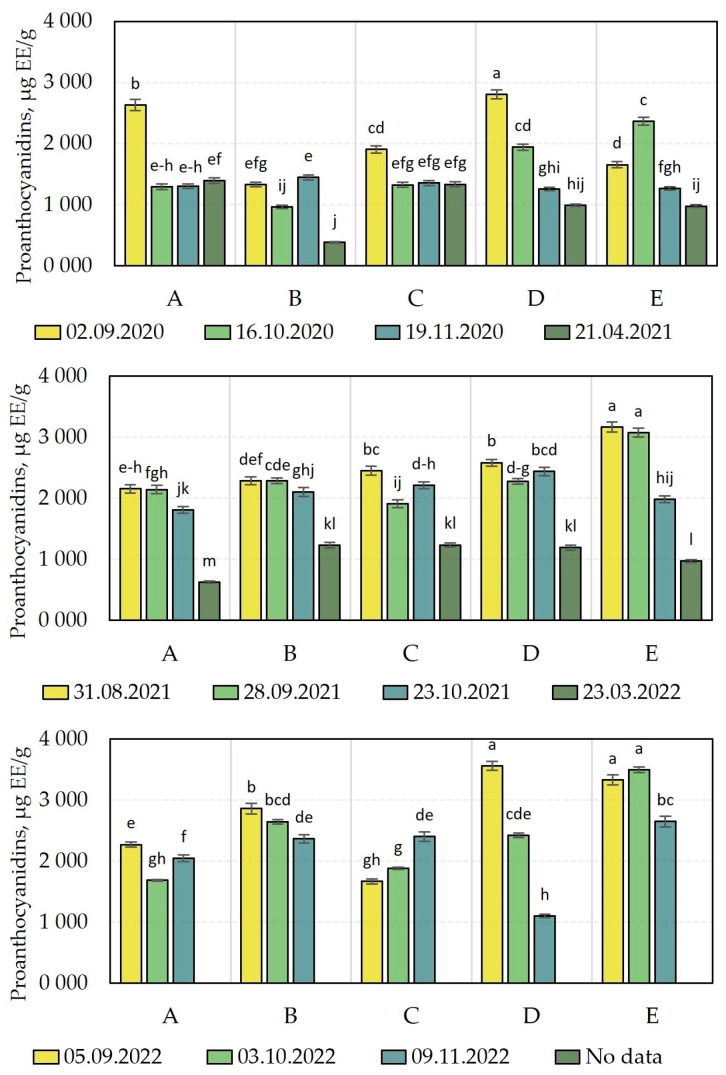
Total proanthocyanidins content in cranberry fruit samples. The wetland habitats (**A**–**E**) of the cranberry fruit sampling sites are described in Table 1. Alphabetic letters indicate the significant difference (*p* < 0.05) between total proanthocyanidins content in the tested cranberry fruits.

We performed a comparative analysis of the total proanthocyanidin content of cranberry fruit samples harvested during the 2020–2022 growing season (Figure 3). We found that the total proanthocyanidin content of the cranberry fruit samples varied: in samples harvested in 2020, it ranged from 378.4 ± 10 µg EE/g to 2803 ± 75 µg EE/g; in samples harvested in 2021, it ranged from 628.3 ± 17 µg EE/g to 3167.7 ± 82 µg EE/g; and in samples harvested in 2022, it ranged from 1096.7 ± 29 µg EE/g to 3557.3 ± 75 µg EE/g (Figure 3). Šedbarė et al. found that the total amount of proanthocyanidins in cranberry fruit samples harvested from the habitats of the Kamanai and Žuvintas State Strict Nature Reserves varied in a similar range—from 919 ± 19 µg EE/g to 3038 ± 137 µg EE/g [15].

The study of the proanthocyanidin content in cranberry fruits harvested in 2020 revealed that the highest levels of proanthocyanidin (2803 ± 75 µg EE/g) were found in cranberry fruits harvested on September 2 from an oligotrophic wetland habitat (site D). Meanwhile, the lowest level of proanthocyanidins (378.4 ± 10 µg EE/g) was established in cranberry fruits harvested from a mesotrophic wetland habitat (site B) in the spring of the following year, after the snowmelt (21 April 2021). The levels of proanthocyanidin of the cranberry fruits harvested from this wetland habitat were not discernibly statistically different from the levels of proanthocyanidin of the fruits harvested from the oligotrophic habitats (sites D and E) in the spring of the following year, after the snowmelt (21 April 2021).

In cranberry fruit samples harvested during the growing season of 2021, the highest total levels of proanthocyanidin compounds were found in cranberry fruit samples harvested from the oligotrophic wetland habitat (site E) from August 31 to September 28 (3167.7 ± 82 µg EE/g and 3075.1 ± 75 µg EE/g, respectively). The lowest total proanthocyanidin content (628.3 ± 17 µg EE/g) was found in cranberry fruit samples harvested from the mesotrophic wetland habitat (site A) in the spring of the following year, after the snowmelt (23 March 2022).

The analysis of the total quantitative composition of proanthocyanidins in cranberry fruit samples harvested in 2022 revealed that the highest total proanthocyanidin content (3557.3 ± 75 µg EE/g) was detected in cranberry fruit samples harvested from an oligotrophic wetland habitat (site C) on November 9. The lowest total proanthocyanidin content (1096.7 ± 29 µg EE/g) established during the growing season of 2022 was found in cranberry fruit samples harvested from the oligotrophic wetland habitat (site D) in November.

The proanthocyanidin content of the fruit samples harvested over the 3-year period varied during ripening (the coefficient of variation was 38.14%) (Figure 3). Cranberry samples harvested in October, November and after snowmelt showed lower levels of procyanidins compared to the total amounts of proanthocyanidins detected in cranberry fruit samples harvested at the end of August and beginning of September. Šedbarė et al. analyzed cranberry fruits harvested from the Kamanai and Žuvintas State Strict Nature Reserves and established that the overall coefficient of variation of proanthocyanidins was 25.98% [15]. The levels of proanthocyanidins established in cranberry fruits harvested from September to mid-October in the Kamanai and Žuvintas habitats varied unevenly, and the variation was influenced by the type of the swamp habitat being investigated [15]. Yu et al. stated that environmental factors (low temperature, UV radiation and exposure to fungal strains) have a significant influence on proanthocyanidin synthesis. These climatic and related other factors affect the intensity of the synthesis of this group of compounds [23].

Analysis of *V. oxycoccos* fruit samples, performed by applying the ultra-performance liquid chromatography-photodiode array-florescence (UPLC-PDA-FLR) method, shows that proanthocyanidins accounted for 63% of the phenolic compounds in cranberry fruit samples [24]. The high concentration of procyanidins in cranberries contributes to the characteristic slightly astringent and bitter taste of the fruit [25]. Proanthocyanidins have antioxidant properties in plants, protecting plants from the formation of free radicals and their damaging effects, as well as improving the ability of the plants to respond to a wide range of environmental stimuli [23].

### 2.3. Analysis of the Qualitative and Quantitative Composition of Flavonols

Flavonols are a specific class of phenols with a wide range of biological effects: antiallergic, antiviral, antitumor, anti-inflammatory, and antioxidant effects [26]. Quercetin established in cranberry fruit has been shown to affect cancer cells in the following organs: bladder [27], breast [28], and ovary [29]. Li et al. found that a flavonol fraction (myricetin and quercetin) isolated from cranberry fruit inhibited the adhesion of *Escherichia coli* to human bladder cells [30].

**Figure 4:**
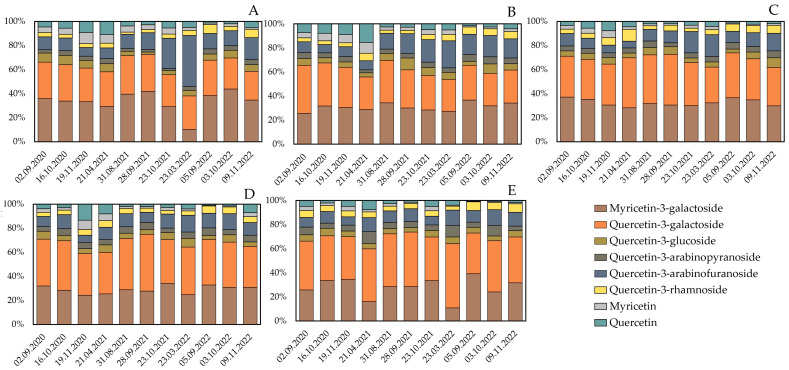
Percentage distribution of identified flavonols in cranberry fruit samples harvested from different wetland habitats. Descriptions of wetland habitats marked with different letters (**A**–**E**) are given in Table 1.

**Figure 5:**
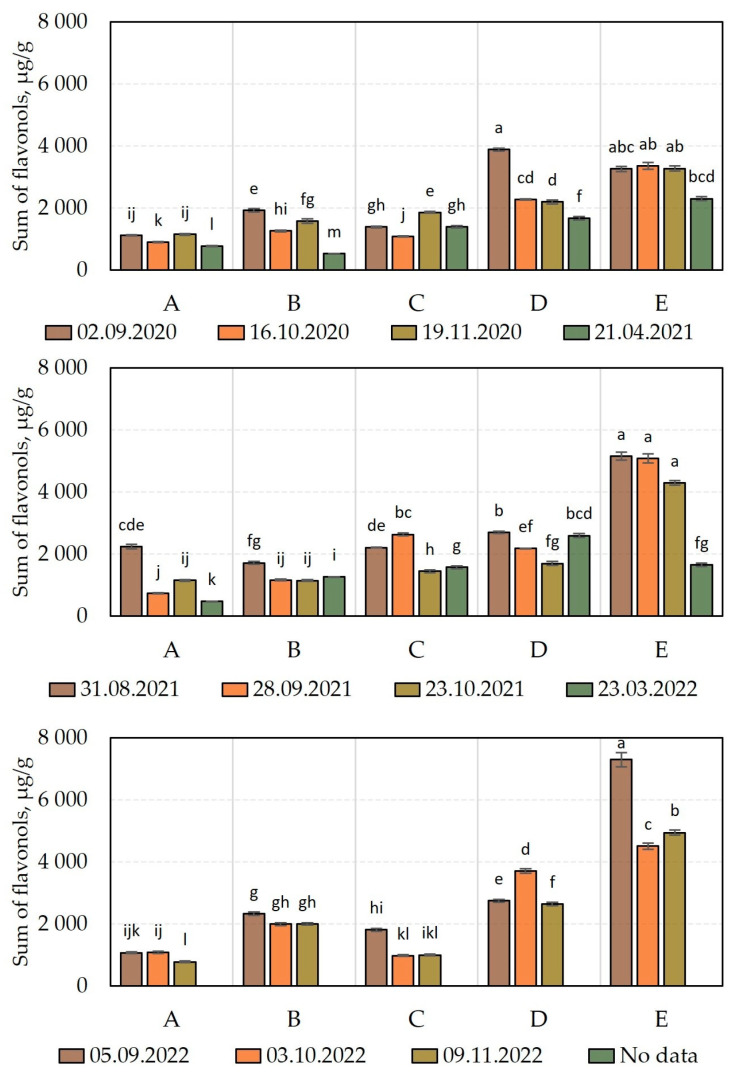
Amount of flavonols in cranberry fruit samples. Alphabetic letters indicate the significant difference (*p* < 0.05) between the sum of flavonols in the tested cranberry fruits. The wetland habitats (**A**–**E**) of the cranberry fruit sampling sites are described in Table 1.

The analysis of the qualitative and quantitative composition of flavonols in cranberry fruit samples harvested during different growing seasons is presented in Figure 4 and Figure 5.

The flavonol composition profile of the bog cranberry (*Vaccinium oxycoccos*) fruits grown under Lithuanian climatic conditions was characteristic of bog cranberry fruits [15]. The flavonol compounds quercetin-3-galactoside (34.9% ± 6.2%) and myricetin-3-galactoside (30.8% ± 6.2%) were the predominant flavonols in the cranberry fruit samples (Figure 4). Other compounds in the flavonol group were found in smaller amounts: quercetin-3-arabinofuranoside (12.3% ± 5.8%), quercetin-3-arabinopyranoside (4.7% ± 1.5%), quercetin-3-glucoside (5.2% ± 1.5%), quercetin-3-rhamnoside (4.6 ± 2.2%), quercetin (4.4% ± 3.1%), and myricetin (3.1% ± 2.2%) (Figure 4). The quantitative composition of flavonols extracted from fruit samples of *V. oxycoccos* in a study by Šedbarė et al. (quercetin-3-galactoside, 31.33% ± 5.10%, myricetin-3-galactoside, 31.38% ± 7.68%, quercetin-3-arabinofuranoside, 12.37% ± 2.41%, quercetin-3-arabinopyranoside, 4.57% ± 1.60%, quercetin-3-glucoside, 4.63% ± 2.84%, quercetin-3-rhamnoside, 4.87 ± 2.03%, quercetin, 2.02% ± 0.66%, and myricetin, 1.82% ± 0.71%) was consistent with the flavonol content of the fruit samples of *V. oxycoccos* found in this study [15].

We carried out a comparative analysis of the total flavonol content of *V. oxycoccos* fruit samples harvested during the growing season of 2020–2022 (Figure 5) and found that the total flavonol content of the cranberry fruit samples varied from 523.18 ± 9 µg/g to 3879.6 ± 39 µg/g in 2020, from 479.6 ± 9 µg/g to 5153.8 ± 129 µg/g in 2021, and from 771.7 ± 23 µg/g to 7291.2 ± 226 µg/g in 2022 (Figure 5). In cranberry fruit samples harvested in 2021 in habitats of the Kamanai and Žuvintas State Strict Nature Reserves, the total flavonol content of cranberries varied less—from 518 ± 16 µg/g to 2811 ± 31 µg/g [15].

The analysis of the quantitative composition of flavonols in cranberry fruit samples harvested in 2020 revealed that the highest total anthocyanin content (3879.6 ± 39 µg/g) was found in cranberry fruit samples harvested on September 2 from an oligotrophic wetland habitat (site D). The levels of flavonols of the cranberry fruits were not discernibly statistically different from those of the fruits harvested from the oligotrophic wetland habitat (site E) on 2 September, 16 October, and 19 November. The lowest total levels of flavonol compounds (523.18 ± 9 µg/g) were established in cranberry fruits harvested from a mesotrophic wetland habitat (site B) in the spring of the following year, after the snowmelt (21 April 2021).

In cranberry fruit samples harvested during the growing season of 2021, the highest total flavonol content (5153.8 ± 129 µg/g) was detected in samples harvested on August 31 from an oligotrophic wetland habitat (site E). The levels of flavonols in the fruits harvested at this site were not discernibly statistically different from the levels of flavonols of the cranberry fruits harvested from the oligotrophic wetland habitat (site E) on September 28 or October 23. The lowest total levels of flavonol compounds (479.6 ± 9 µg/g) were established in cranberry fruits harvested from a mesotrophic wetland habitat (site A) in the spring of the following year, after the snowmelt (23 March 2022).

The study of the flavonol content in cranberry fruits harvested in 2022 showed that the maximum observed total levels of flavonols (7291.2 ± 226 µg/g) were established in cranberry fruits harvested on September 5 from an oligotrophic wetland habitat (site E). Meanwhile, the lowest total levels of flavonols (518 ± 16 µg/g) during the growing season of 2022 was established in cranberry fruits harvested from a mesotrophic wetland habitat (site A) in November. The levels of flavonols of the fruits were not discernibly statistically different from those of the cranberry fruits harvested on October 3 or November 9 from the oligotrophic wetland habitat (site C).

In the oligotrophic wetland-type habitats (sites D and E), cranberry fruit samples harvested between 2020 and 2022 had a higher flavonol content than those harvested from the other sites did, and we found that flavonol content tended to increase over the years (2020 < 2021 < 2022). As the snow melted, the flavonol content of the cranberry fruit samples harvested either showed a statistically discernible decrease or remained unchanged. Šedbarė et al. investigated cranberry fruit samples harvested at the beginning of September and in October in 12 wetland habitats of the Kamanai and Žuvintas State Strict Nature Reserves. The comparative analysis showed that the flavonol content of cranberry fruit samples harvested from five oligotrophic habitats was higher than that of samples harvested from five eutrophic or mesotrophic wetland habitats [15]. Albert et al. suggested that flavonol biosynthesis is influenced by light in plants, vegetative organs, developing flowers and fruits, and covering tissues (including UVA and UVB) [31]. In oligotrophic wetland habitats, cranberry groves are located in more open areas. Thus, direct exposure to light can affect the biosynthesis of bioactive compounds in cranberry fruit tissues. Nutrient deficiencies in oligotrophic wetland-type habitats may lead to increased plant stress and the increased synthesis of flavonol compounds [32,33]. In the analysis of cranberry fruit samples harvested at the end of August and the beginning of September in the years 2020–2022 for the determination of the correlation between weight per berry and flavonol content, we found that the flavonol content of the cranberry fruit samples negatively correlated with the weight per berry, the correlation coefficient being −0.828 (*p* < 0.05). Flavonols are more abundant in the skin of fruit. This is why, in the presence of unfavorable abiotic conditions, smaller berries have higher flavonol content [34,35].

### 2.4. Analysis of the Quantitative Composition of Chlorogenic Acid

Phenolic carboxylic acids are widespread in angiosperm plants [36]. They have a wide range of biological effects, and thus there is ongoing research into their identification [37]. Chlorogenic acid has been found to predominate in cranberry fruit plant material and has been shown to enhance the sensory and biological properties of the berries (reducing obesity, dyslipidemia, and arterial blood pressure) [25,38].

**Figure 6:**
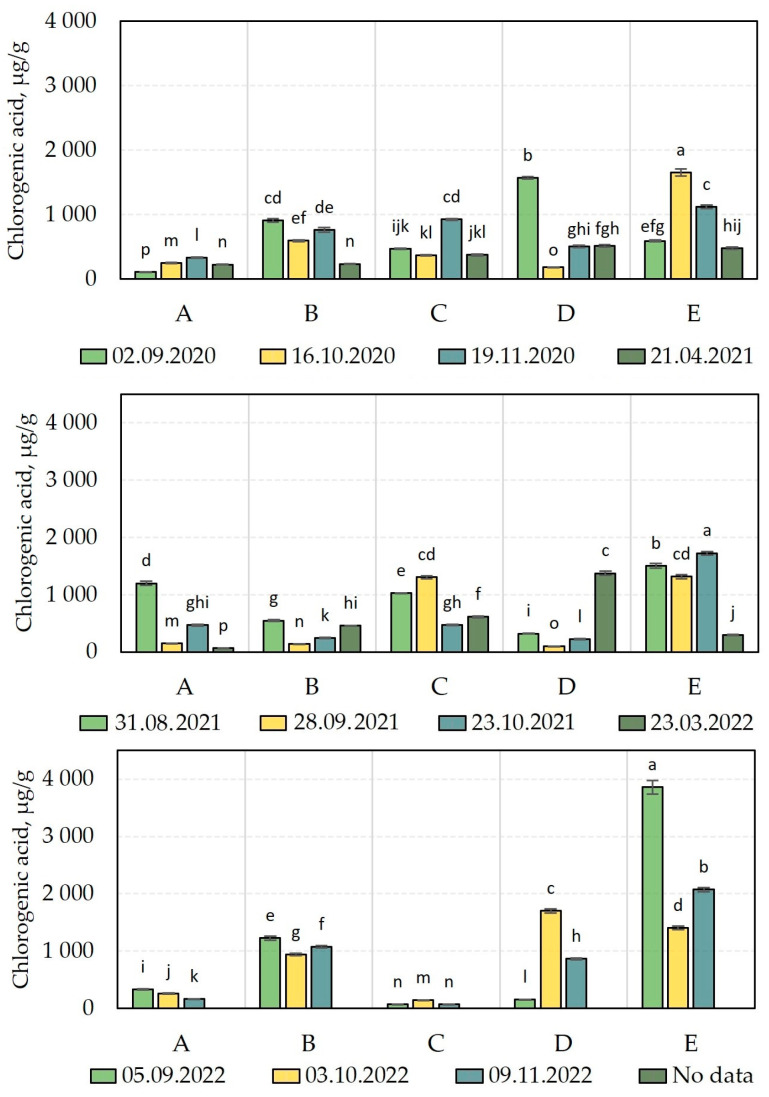
Chlorogenic acid content in cranberry fruit samples. The wetland habitats (**A**–**E**) of the cranberry fruit sampling sites are described in Table 1. Alphabetic letters indicate the significant difference (*p* < 0.05) between the chlorogenic acid content in the tested cranberry fruits.

We carried out a comparative analysis of the chlorogenic acid content of *V. oxycoccos* fruit samples harvested during the plant growing season of 2020–2022 (Figure 6). We found that the amount of chlorogenic acid varied in the fruit samples harvested: 108.8 ± 2 µg/g to 1648.6 ± 53 µg/g in the growing season of 2020, 68.0 ± 1 µg/g to 1722.6 ± 30 µg/g in the growing season of 2021, and 71.2 ± 2 µg/g to 3858.2 ± 119 µg/g in the growing season of 2022 (Figure 6). The coefficient of variation in the cranberry fruit samples tested was 93.85%. Šedbarė et al. found that the amount of chlorogenic acid in bog cranberry samples varied from 17 ± 0.4 µg/g to 1224 ± 41 µg/g, with a coefficient of variation of 113.9% [15].

The assay of the quantitative composition of chlorogenic acid in cranberry fruits harvested in 2020 revealed that the maximum observed level of chlorogenic acid (1648.6 ± 53 µg/g) was established in cranberry fruit samples harvested on October 3 from an oligotrophic wetland habitat (site E). On the contrary, the lowest level of chlorogenic acid (108.8 ± 2 µg/g) was established in cranberry fruits harvested from a mesotrophic wetland habitat (site A) in early September.

The evaluation of the quantitative composition of chlorogenic acid in cranberry fruits harvested in 2021 revealed that the maximum level of chlorogenic acid (1722.6 ± 30 µg/g) was established in cranberry fruits harvested from an oligotrophic wetland habitat (site E) in October. On the contrary, the lowest level of chlorogenic acid (68.0 ± 1 µg/g) was established in cranberry fruits harvested from a mesotrophic wetland habitat (site A) in the spring of the following year, after the snowmelt (23 March 2022).

In cranberry fruit samples harvested during the growing season of 2022, the highest amount of chlorogenic acid (3858.2 ± 119 µg/g) was detected in cranberry fruit samples harvested from an oligotrophic wetland habitat (site E) in early September. The lowest levels of chlorogenic acid (71.2 ± 2 µg/g and 74.5 ± 2 µg/g) were found in cranberry fruit samples harvested from an oligotrophic wetland habitat (site C) in September and November, accordingly.

Studies on the quantitative composition of chlorogenic acid in cranberry raw material allowed the determination of the content of chlorogenic acid in the raw material samples and its trends during fruit ripening over a 3-year period (2020–2022). Cranberry fruit samples harvested in different years in an oligotrophic wetland habitat (site E) showed the highest levels of chlorogenic acid. In other wetland habitats, the level of chlorogenic acid established in cranberries varied between multiple points of different seasons. Šedbarė et al. carried out a quantitative analysis of chlorogenic acid and established that cranberries harvested from one mesotrophic wetland habitat and one oligotrophic wetland habitat were found to contain high levels of chlorogenic acid [15]. Soviguidi et al. argue that during plant evolution, exposure to deleterious climatic environmental factors (e.g., drought, soil salinity, ionic toxicity, nutrient deficiency, and/or UV radiation) allows genes responsible for the chlorogenic acid biosynthesis-promoting effect to be established. The antioxidant properties of chlorogenic acid improve the plant’s ability to cope with adverse climatic factors [39].

### 2.5. Analysis of the Qualitative and Quantitative Composition of Triterpenoids

The outer surface of cranberries is covered with a waxy layer, which performs a protective function against water evaporation, microorganisms, UV rays and temperature changes. [40]. Triterpene compounds comprise about 50% of the waxy layer of cranberry fruit [41,42]. Triterpene compounds established in cranberries have antioxidant [43] and anti-inflammatory [44] properties. Murphy et al. found that triterpene compounds detected in cranberry fruit samples inhibited the growth of prostate, cervical, lung, breast, and colon tumors, as well as leukemia cells, in in vitro models [45].

**Figure 7:**
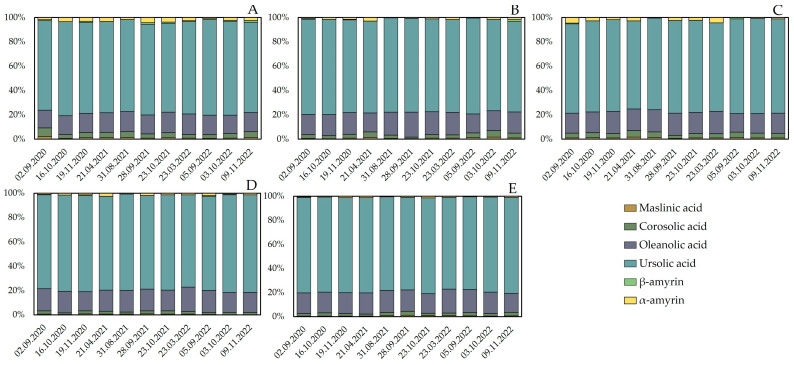
Percentage distribution of identified triterpenoids in cranberry fruit samples harvested from different wetland habitats. Descriptions of wetland habitats marked with different letters (**A**–**E**) are given in Table 1.

The phytochemical profile of triterpenoids found in bog cranberry fruit samples harvested from wetland habitats in the Čepkeliai State Strict Nature Reserve was characteristic of bog cranberry fruit (Figure 7) [46].

Ursolic acid was the predominant acid in the cranberry fruit samples, accounting for 76.8% ± 2.0% of the detected triterpenoids (Figure 7). Klavins et al. confirm that ursolic acid is the predominant triterpenoid in cranberry fruit [42]. The amount of oleanolic acid in cranberry fruit samples was 17.12% ± 1.3%. For the other triterpene compounds, lower levels were detected: the amount of maslinic acid was 0.83% ± 0.4%, the amount of corosolic acid was 3.2% ± 1.3%, the amount of α-amyrin was 1.65% ± 1.1%, and the amount of β-amyrin was 0.28% ± 0.5% (Figure 7). In a study by Šedbarė et al., the detected quantitative composition of triterpenoids in *V. oxycoccos* fruit samples (ursolic acid, 76.24% ± 1.91%, oleanolic acid, 17.44% ± 1.17%, maslinic acid, 0.91% ± 0.48%, corosolic acid, 3.32% ± 1.49%, α-amyrin, 1.98% ± 1.39%, and β-amyrin, 0.10% ± 0.24%) was consistent with the triterpenoid composition established in fruit samples of *V. oxycoccos* in the present study [15].

**Figure 8:**
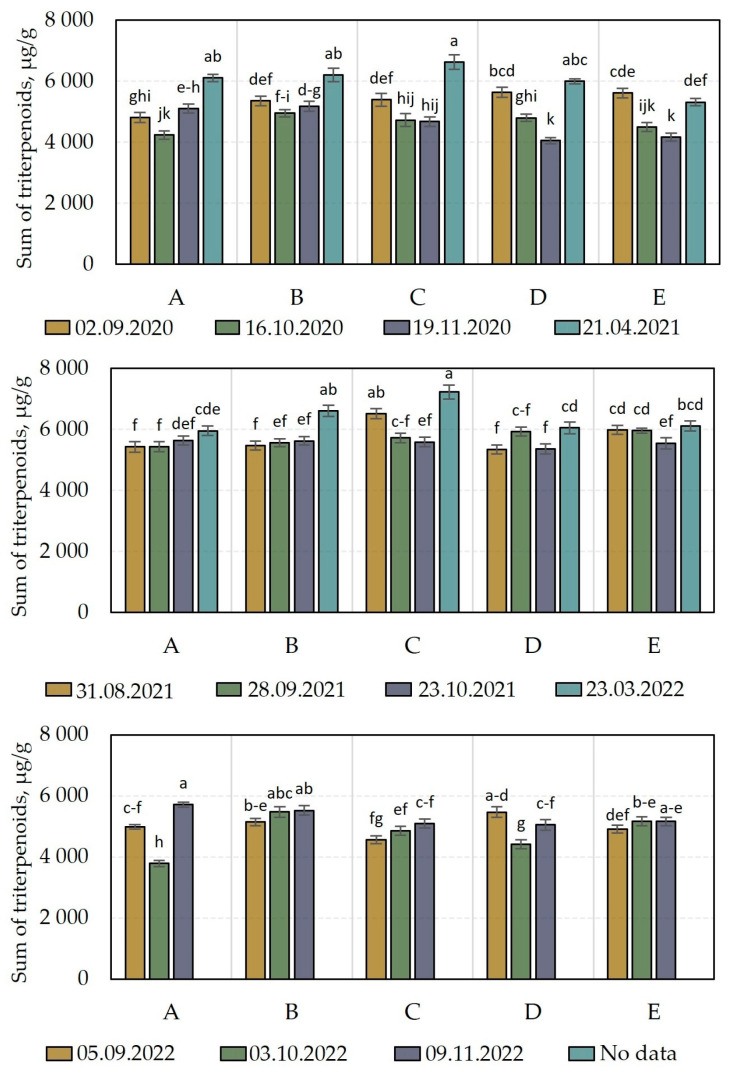
Amount of triterpenoids in cranberry fruit samples. The wetland habitats (**A**–**E**) of the cranberry fruit sampling sites are described in Table 1. Alphabetic letters indicate the significant difference (*p* < 0.05) between the sum of triterpenoids in the tested cranberry fruits.

We carried out a comparative analysis of the total amounts of triterpenoids in *V. oxycoccos* fruit samples harvested during the 2020–2022 plant growing season (Figure 8). We found that the total amounts of triterpene compounds varied in the fruit samples harvested: during the 2020 growing season, they ranged from 4054.9 ± 101 µg/g to 6628.8 ± 238 µg/g; during the 2021 growing season, they ranged from 5346.1 ± 139 µg/g to 7226.9 ± 224 µg/g; and during the 2022 growing season, they varied from 3780.8 ± 98 µg/g to 5708.0 ± 80 µg/g (Figure 8). In a study by Šedbarė et al., the total amounts of triterpene compounds found in cranberry fruit samples harvested from the Kamanai and Žuvintas State Strict Nature Reserves were found to be in similar ranges, from 4060 ± 122 µg/g to 6542 ± 157 µg/g [15].

In cranberry fruit samples harvested during the 2020 growing season, the highest triterpenoid content (6628.8 ± 238 µg/g) was detected in the samples harvested from the oligotrophic wetland habitat (site C) in the spring of the following year, after the snowmelt (21 April 2021). The levels of triterpene compounds in the fruits harvested from this habitat were not discernibly statistically different from the total levels of triterpene compounds of cranberry fruits harvested from the oligotrophic wetland habitat (site D) or the mesotrophic wetland habitat (sites A and B) in the spring of the following year, after the snowmelt (21 April 2021). The lowest levels of triterpenoids (4054.9 ± 101 µg/g) were established in the cranberry fruits harvested from the oligotrophic wetland habitat (site D) in November. These were not discernibly statistically different from the levels of triterpene compounds established in the cranberry fruits harvested from the oligotrophic wetland habitat (site E) in November.

The study of the composition of triterpenoids in cranberry fruits harvested in 2021 showed that the maximum observed levels of triterpene compounds (7226.9 ± 224 µg/g) were established in cranberry fruits harvested from an oligotrophic wetland habitat (site C) in the spring of the following year, after the snowmelt (23 March 2022). The levels of triterpene compounds in the cranberry fruits harvested from this habitat were not discernibly statistically different from the total levels of triterpenes in the fruits harvested from the mesotrophic wetland habitat (site B) the following spring on 23 March 2022 or from the oligotrophic wetland habitat (site C) on 31 August 2021. The lowest total level of triterpenes (5346.1 ± 139 µg/g) was established in cranberry fruits harvested on August 31 from an oligotrophic wetland habitat (site D). The levels of triterpenes in the fruits were not discernibly statistically different (*p* > 0.05) from those established in most of the cranberry fruits harvested in August–November (Figure 8).

The analysis of the quantitative composition of triterpene compounds in cranberry fruit samples harvested in 2022 revealed that the maximum observed total amount of triterpene compounds (5708.0 ± 80 µg/g) was found in cranberry fruit samples harvested in November from a mesotrophic wetland habitat (site A). The quantitative composition of triterpene compounds detected in the fruit samples harvested from this habitat was not discernibly statistically different from that detected in the cranberry fruit samples harvested from the mesotrophic wetland habitat (site B) in October and November, from the oligotrophic wetland habitat (site D) in August, or from the oligotrophic wetland habitat (site E) in November. The lowest triterpenoid content (3780.8 ± 98 µg/g) was detected in cranberry fruit samples harvested on August 31 from a mesotrophic wetland habitat (site A).

The amount of triterpene compounds in cranberry fruit samples harvested from the Čepkeliai State Strict Nature Reserve wetland habitats in August–November in different years (2020–2022) varied less than the content of phenolic compounds did (the coefficient of variation was 10.76%). Šedbarė et al. in their study found a similar variation (10.52%) in cranberry fruit samples harvested at the Kamanai and Žuvintas State Strict Nature Reserves [15].

The Čepkeliai State Strict Nature Reserve is an area on the south-eastern edge of Lithuania [5]. The climate of the area is characterized by greater seasonal temperature contrasts compared to other parts of Lithuania due to the sandy wetland-type areas that easily warm up and easily lose their heat [5,47]. The continental climate of the Čepkeliai State Strict Nature Reserve, with its warmer summers and colder winters, also results in a longer-lasting snow cover, which is why, during the study period in 2021 and 2022, the snow melted in spring [47]. An increase in the amount of triterpene compounds was detected in cranberry fruit samples harvested in spring, after the snowmelt. Biswas and Dwivedi argue that abiotic stress factors (nutrient starvation, light, and temperature) affect the qualitative and quantitative composition of triterpene compounds, and that the biosynthesis of triterpenes is enhanced by external stimuli (pathogen attack, cold, and/or drought) [48]. The results of the studies on the variation of triterpenoid compounds suggest that the biosynthesis of triterpenoids in cranberry fruits is more intense in the spring when the plants are exposed to the climatic factors typical of the environment.

### 2.6. Comparative Analysis of the Phytochemical Composition of Cranberry Fruit Samples for the Period of 2020–2022

**Figure 9:**
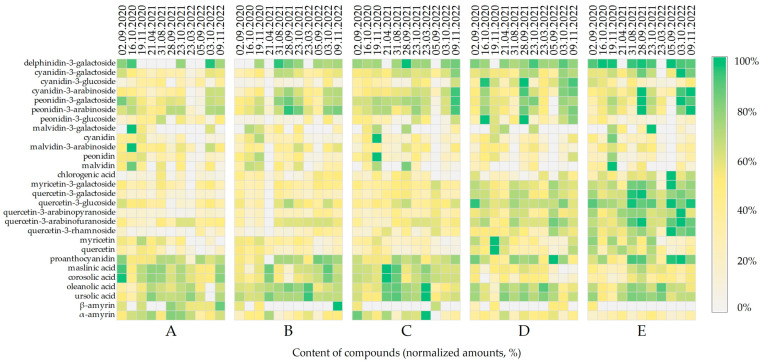
Heat map of individual constituent content (%) in cranberry fruits according to their wetland habitat. The wetland habitats (**A**–**E**) of the cranberry fruit sampling sites are described in Table 1.

Changes in the composition of biologically active compounds during cranberry fruit development and ripening are determined using genetic factors [15], environmental conditions (soil composition, temperature, humidity, and light) [49], and the stage of the ripeness of the fruit [50]. The qualitative and quantitative composition of phenolic compounds and triterpene compounds in cranberry fruit samples varied over the 3-year study period and during the ripening stage. In order to systematize and summarize the obtained results, a comparative analysis of the trends in the accumulation of the qualitative and quantitative composition of biologically active compounds was carried out by means of heatmap visualization (Figure 9).

We established trends in terms of the higher or lower levels of some compounds in cranberries harvested from different wetland habitats. The model in Figure 9 shows that cranberries harvested from oligotrophic wetland habitats (sites E and D) contained higher levels of anthocyanins, anthocyanidins, flavonol glycosides, and proanthocyanidins. Cranberries harvested from mesotrophic wetland habitats (sites A and B) and oligotrophic wetland habitats (Site C) contained higher levels of triterpene compounds, among which maslinic and corosolic acids predominated.

The oligotrophic habitats (sites D and E) are located in the higher part of the Čepkeliai wetland, where lower plants such as *Sphagnum magellanicum*, *Sphagnum angustifolium*, *Polytrichum strictum*, and *Sphagnum capillifolium* grow, which means that cranberries are exposed to direct sunlight for longer periods during the day. This creates favorable conditions for the biosynthesis of biologically active compounds in the fruit and influences the ripening process. Zhou et al. found that exposure to sunlight increased the total anthocyanin content of cranberry fruit by 75.3% in 24 h and by 87.2% in 48 h compared to a control sample kept in the dark [51]. Daryanavard et al. pointed out that flavonols protect plants against changes in environmental conditions and UV radiation, and that the biosynthesis of flavonol group compounds is enhanced by exposure to adverse abiotic environmental conditions [52].

Cranberry fruits of smaller weigh grew in oligotrophic-type (sites D and E) bogs. Consequently, smaller-weigh fruits accumulated a higher concentration of biologically active compounds. Correlation analysis revealed a significant negative correlation between fruit size and flavonol content (−828, *p* < 0.05) and proanthocyanidin content (−0.642, *p* < 0.05). Similar results have been obtained in studies of other fruits of the genus *Vaccinium* [53,54]. No correlation was found between fruit weight and anthocyanin or triterpenoid content. No correlation was found between the pH value and the amount of identified compounds. Vorsa and Zalapa indicate that quantification of flavonol and proanthocyanidin content of cranberry fruit is based on a per whole-fruit fresh weight basis, and are not based on the concentrations of specific tissue, e.g., epidermis versus flesh [55]. In addition, the authors suggest that larger cranberry fruit might be expected to have a lower epidermis-to-pulp ratio, resulting in smaller cranberry fruit having higher flavonoid content [55].

**Figure 10:**
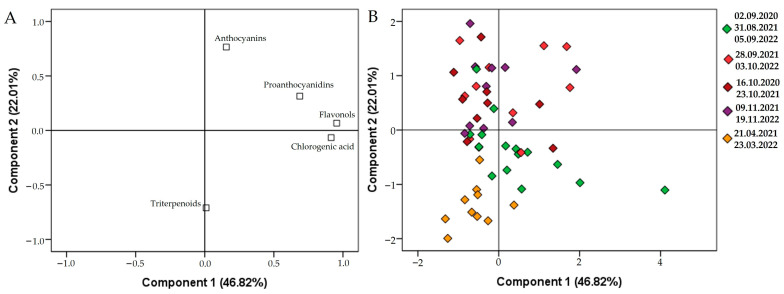
Principal component analysis loading (**A**) and score (**B**) plots of different cranberry samples.

Principal component analysis was carried out to assess the content variation of triterpene compounds, flavonols, chlorogenic acid, proanthocyanidins, and anthocyanins established in cranberries during the ripening of the berries in the period of 2020–2022 (Figure 10). Principal component I explained 46.82% of the total variance of the data and had a discernible correlation with the anthocyanidins (0.156), proanthocyanidins (0.687), chlorogenic acid (0.915), and flavonols (0.954). Principal component II explained 22.01% of the total variance of the data and had a discernible correlation with the triterpene compounds (−0.710), proanthocyanidins (0.315), and anthocyanidins (0.765).

Most of the cranberry fruit samples harvested at the end of August and the beginning of September were located in the negative area of principal component II. The harvested samples showed higher levels of proanthocyanidins and flavonols and low levels of anthocyanins. In the coordinate plane, the points of the cranberry fruit samples harvested in late September, October, and November were scattered in the positive area of principal component II. These samples contained the highest levels of anthocyanins, while the levels of flavonols, chlorogenic acid, and proanthocyanidins also remained high. The most significant changes in the quantitative composition of anthocyanins and anthocyanidins were found during fruit ripening [56]. Karppinen et al. reported that fruit ripening increases the transcription levels of enzymes involved in anthocyanin biosynthesis: UDP-glucose flavonoid 3-*O*-glucosyltransferase (UFGT), dihydroflavonol 4-reductase (DFR), anthocyanidin synthase (ANS), and chalcone synthase (CHS). In turn, this increases the anthocyanin content of the fruit [57]. The anthocyanin content of bog cranberry fruits increased on average by a factor ranging from 2 to 6 between the end of August and mid-November. The assessment of the ripening stage of the fruit, which determines the quantitative composition of the anthocyanin group of compounds in cranberry plant material, is important for the timing of harvesting in cranberry habitats.

The samples harvested in spring, after the snowmelt, were located in the area of negative values of principal components I and II. These cranberry fruit samples showed a decrease in the amounts of anthocyanins, flavonols, proanthocyanidins, and chlorogenic acid, while their levels of triterpene compounds were the highest.

As the quantitative composition of biologically active compounds changes during the ripening stage of cranberries, it is important to perform studies on the phytochemical composition of cranberry raw material in order to provide consumers with cranberry fruit raw material with a high commercial value and a known qualitative and quantitative composition. The highest levels of anthocyanins were found in samples of ripe cranberries. Anthocyanin levels varied depending on the period of fruit preparation and the characteristics of the types of habitats. The detected quantitative changes in flavonols, proanthocyanidins, and chlorogenic acid were lower than those in anthocyanins. Routine studies on the qualitative and quantitative composition of biologically active compounds in cranberry raw material would allow for the monitoring of the composition of cranberry raw material and its preparations and the prediction of their biological effects [58,59].

## 3. Materials and Methods

### 3.1. Chemicals

All the chemicals used in the experiments were of an analytical grade. Cyanidin chloride, cyanidin-3-galactoside, cyanidin-3-glucoside, cyanidin-3-arabinoside, peonidin chloride, peonidin-3-arabinoside, peonidin-3-glucoside, peonidin-3-galactoside, myricetin-3-galactoside, malvidin-3-galactoside, malvidin chloride, malvidin-3-arabinoside, delphinidin-3-galactoside (Extrasynthese, Genay, France), quercetin-3-*O*-glucoside (Biochemistry, Buchs, Switzerland), quercetin-3-galactoside, quercetin (Carl Roth, Karlsruhe, Germany), myricetin, quercetin-3-rhamnoside, quercetin-3-α-l-arabinofuranoside, quercetin-3-α-l-arabinopyranoside, oleanolic acid, ursolic acid, α-Amyrin, β-Amyrin, corosolic acid, and maslinic acid (Sigma-Aldrich, Steinheim, Germany) were used as standards for the identification of the compounds. Acetone, methanol, acetonitrile, hydrochloric acid, and 4-(Dimethylamino) cinnamaldehyde) were purchased from Sigma-Aldrich (Steinheim, Germany). Ethanol 96% (*v*/*v*) was purchased from AB Stumbras (Kaunas, Lithuania). Formic acid was bought from Merck (Darmstadt, Germany).

### 3.2. Plant Material

Bog cranberries (*Vaccinium oxycoccos* L.) were harvested from five different places in the territory of the Čepkeliai State Strict Nature Reserve in 2020–2022 (Table 1). The cranberry sampling dates were 2 September 2020, 16 October 2020, 19 November 2020, 21 April 2021 (after the snowmelt), 31 August 2021, 28 September 2021, 23 October 2021, 23 March 2022 (after the snowmelt), 5 September 2022, 5 October 2022, and 9 November 2022.

Data on cranberry fruit weight and pH characteristics of wetland habitats are presented in Table 2. Wetland water acidity (pH) was measured in habitats. Berries were harvested 5 times in 0.25 m^2^ frames placed in random areas of wetland habitats. Afterwards, the berries were counted and weighed and the average values for 1 berry were calculated.

Samples of cranberry fruits were cut in half after harvesting and frozen at −60 °C in a freezer (CVF330/86, ClimasLab SL, Barcelona, Spain). Freeze-drying was performed in a lyophilizer (Zirbus Technology GmbH, Bad Grund, Germany). The samples were freeze-dried at a pressure of 0.01 mbar and at a condenser temperature of −85 °C. The dried samples were ground using a Retsch GM 200 electric mill (Retsch GmbH, Hahn, Germany). The moisture content of all cranberry fruit samples was measured using the European Pharmacopoeia method [60].

### 3.3. Sample Preparation by Extraction

An ultrasonic device (Elmasonic P, Elma Schmidbauer GmbH, Singen, Germany) was used for an ultrasound-assisted extraction procedure. For the extraction of anthocyanins, proanthocyanidins, and flavonols, 1 g of lyophilized cranberry fruit powder was mixed with 20 mL of 70% ethanol acidified with 1% hydrochloric acid (*v*/*v*). When the procedure was performed using an effective ultrasonic power of 565 W and an ultrasonic frequency of 80 kHz at room temperature, the duration of ultrasonic extraction was 15 min. After the extraction, the prepared cranberry extracts were filtered into a 20 mL volumetric flask. Three extraction replicates were performed for each sample. The prepared cranberry extracts were stored at −20 °C for further analyses.

For the extraction of triterpenoids, 1 g of lyophilized cranberry fruit powder was mixed with 10 mL of 100% (*v*/*v*) acetone. When the procedure was performed using an effective ultrasonic power of 1130 W and an ultrasonic frequency of 80 kHz from 22 ± 1 °C to 60 ± 1 °C, the duration of ultrasonic extraction was 60 min. After the extraction, the prepared cranberry extracts were filtered into a 10 mL volumetric flask. Three extraction replicates were performed for each sample. The prepared cranberry extracts were stored at −20 °C for further analyses.

### 3.4. Spectrophotometric Studies

A spectrophotometer (M550 UV/Vis, Spectronic CamSpec, Garforth, UK) was used for spectrophotometry analysis. Proanthocyanidins were measured with DMCA (4-(dimethylamino)cinnamaldehyde) [61]. Twenty microliters of the cranberry fruit extract were mixed with 3 mL of the DMCA reagent (0.1% DMCA (*m*/*v*) in ethanol–hydrochloric acid at 9:1 (*v*/*v*)). The blank was the DMCA solution without the extract. Optical density was measured with a spectrophotometer at 640 nm after 5 min. A standard curve was established beforehand with a (–)-epicatechin (µg/g (–)-epicatechin equivalent (EE) dry weight and a calibration equation of 𝑦 = 0.7021𝑥 + 0.0138. R^2^ = 0.9994.

### 3.5. UPLC Analysis

Phenolic compounds and triterpenoids were identified using a Waters ACQUITY UPLC chromatograph (Milford, MA, USA) equipped with a photodiode array detector (ACQUITY UPLC PDA eλ, Milford, MA, USA). The compounds were separated on a reverse-phase, 100 × 2.1 mm, 1.7 µm-particle-size ACE C18 analytical column (An Avantor ACE, ACT, Aberdeen, UK). Samples of the three cranberry fruit extracts were filtered through a filter with a pore size of 0.22 µm (Carl Roth GmbH, Karlsruhe, Germany) into vials and injected into the UPLC system with a 1 μL volume injection. The data were collected using the Empower 3 (Waters) Software. The quantity of each compound was calculated using the calibration curve of the external standards. Concentrations were expressed in µg per g of dry weight.

The analysis of anthocyanins and anthocyanidins was performed using the validated technique previously described by Vilkickyte et al. [62]. The column was operated at 30 °C with the elution solvents A (100% acetonitrile) and B (10% formic acid in water) and a flow rate of 0.5 mL/min. The gradient was as follows: 0–2 min, 95% B; 2–7 min, 91% B; 7–9 min, 88% B; 9–10 min, 75% B; 10–10.5 min, 20% B; 10.5–11 min, 20% B; and, finally, reconditioning of the column (11–12 min, 95% B). Anthocyanins were detected at a wavelength of 520 nm. The identification of anthocyanins in cranberry fruits samples was achieved by comparing retention times and UV/Vis spectra with the retention times and spectra of the external standards.

The analysis of flavonols and chlorogenic acid was performed using the validated technique previously described by Urbstaite et al. [63]. The column was operated at 30 °C with the elution solvents A (100% acetonitrile) and B (0.1% formic acid in water) and a flow rate of 0.5 mL/min. The gradient was as follows: 0 min, 95% B; 0–1 min, 88% B; 1–3 min, 88% B; 3–4 min, 87% B; 4–9 min, 75% B; 9–10.5 min, 70% B; 10.5–12 min, 70% B; 12–12.5 min, 10% B; 12.5–13 min, 10% B; 13–13.5 min, 95% B; and 13.5–14.5 min, 95% B. The next injection was delayed for 2 min to allow the eluents in the column to equilibrate. Flavonols were detected at a wavelength of 360 nm, and chlorogenic acid was detected at a wavelength of 330 nm. The identification of flavonols and chlorogenic acid in cranberry fruits samples was achieved by comparing retention times and UV/Vis spectra with the retention times and spectra of the external standards.

Triterpenoid analysis was performed using the validated technique previously described by Sedbare et al. [46]. The column was operated at 25 °C with elution solvents A (100% methanol) and B (0.1% formic acid in water) and a flow rate of 0.2 mL/min. The gradient was as follows: 0 min, 8% B; 0–8 min, 3% B; 8–9 min, 2% B; 9–29.5 min, 2% B; and, finally, reconditioning of the column (29.5–30 min, 8% B). The next injection was delayed for 10 min to allow the eluents in the column to equilibrate. Triterpene compounds were detected at a wavelength of 205 nm.

### 3.6. Data Analysis

The data were statistically analyzed using SPSS Statistics 21 (IBM, Armonk, NY, USA) and Microsoft Excel 2016 (Microsoft, Redmond, DC, USA) with a 5% significance level for variation analysis. The Kruskal–Wallis one-way ANOVA test was used to determine differences in the content of the identified compounds between cranberry samples. The data of the quantitative analysis were analyzed by applying principal component analysis (PCA). The means and standard deviations of the three independent evaluations were calculated.

## 4. Conclusions

Studies on the qualitative and quantitative composition of bog cranberry fruit samples provide new insights into the variation in the composition of anthocyanins, proanthocyanidins, chlorogenic acid, triterpenic compounds, and flavonols in cranberry fruit. These data are valuable for determining the optimum time for cranberry harvesting, for investigating the influence of different wetland habitats on the quantitative composition of bioactive compounds, for assessing the quality of cranberry fruit, and for evaluating the use of quality cranberry fruit raw material for food and health purposes.

The phytochemical composition of the cranberry fruits growing in high wetlands varied over the three-year period (2020–2022), but the overall patterns of change in bioactive compounds showed that the highest levels of bioactive compounds were concentrated in the oligotrophic habitats (sites D and E). These are located in the higher part of the Čepkeliai marsh, where the plants are lower and where they are exposed to direct sunlight and climatic conditions that enhance the biosynthesis of flavonoids. The most significant changes in anthocyanin content during ripening, with peak levels reached in October–November (when the fruit are fully ripe), resulted in a balanced content of bioactive compounds in the cranberry fruit samples.

## Figures and Tables

**Table 1:** Description of cranberry fruit harvesting areas.

Site	Coordinates	Altitude (m)	Characteristics of the Wetland Environment
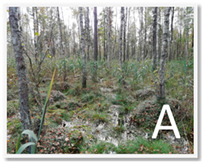	53°59′3.13″ N, 24°26′49.37″ E	130.7	Mesotrophic habitat; ass. Betuletum pubescentis (Hueck 29) Tx. 37. Dominant plants: *Betula pubescens*, *Phragmites australis*, *Carex rostrata*, *Sphagnum palustre.*
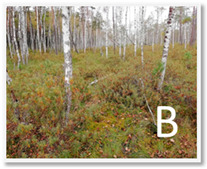	53°59′7.31” N, 24°26′49″ E	131.7	Mesotrophic habitat; ass. Betuletum pubescentis (Hueck 29) Tx. 37. Dominant plants: *Betula pubescens*, *Calla palustris Eriophorum vaginatum*, *Oxycoccus palustris*, *Sphagnum cuspidatum.*
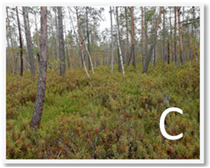	53°59′9.85″ N, 24°26′53.7″ E	132.0	Oligotrophic habitat; Ass. Ledo-Pinetum Tx.55. Dominant plants: *Pinus sylvestris f. Litwinowii*, *Rhododendron tomentosum*, *Eriophorum vaginatum*, *Oxycoccus palustris*, *Sphagnum magellanicum s. l.*
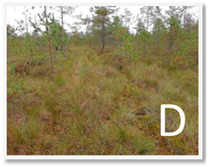	53°59′18.35″ N, 24°27′7.48″ E	132.3	Oligotrophic habitat; ass. Sphagnetum magellanici (Malcuit 1929) Kästner et Flössner 1933. Dominant plants: *Pinus sylvestris f. Wilkommii*, *Calluna vulgaris*, *Oxycoccus palustris*, *Sphagnum magellanicum s.l.*, *Sphagnum angustifolium.*
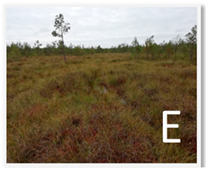	53°59′37.86″ N, 24°27′44.93″ E	132.6	Oligotrophic habitats; ass. Sphagnetum magellanici (Malcuit 1929) Kästner et Flössner 1933. Dominant plants: *Oxycoccus palustris*, *Eriophorum vaginatum*, *Sphagnum capillifolium ir Polytrichum strictum.*

**Table 2:** Data on cranberry fruit mass and pH characteristics of wetland habitats.

Site	Average Weight of 1 Berry 2020	Average Weight of 1 Berry 2021	Average Weight of 1 Berry 2022	pH 2020	pH 2021	pH 2022
A	0.42	0.64	0.66	4.27	4.05	3.78
B	0.54	0.66	0.45	4.42	3.93	3.66
C	0.62	0.50	0.60	4.27	3.92	3.50
D	0.35	0.34	0.36	3.68	3.94	3.63
E	0.37	0.32	0.20	3.60	4.28	4.36

## Data Availability

All data generated during this study are included in this article.

## Sample Availability

Not available.
